# *Tetris* Is a Foldback Transposon that Provided the Building Blocks for an Emerging Satellite DNA of *Drosophila virilis*

**DOI:** 10.1093/gbe/evu108

**Published:** 2014-05-24

**Authors:** Guilherme B. Dias, Marta Svartman, Alejandra Delprat, Alfredo Ruiz, Gustavo C.S. Kuhn

**Affiliations:** ^1^Departamento de Biologia Geral, Universidade Federal de Minas Gerais, Belo Horizonte, MG, Brazil; ^2^Departament de Genètica i de Microbiologia, Universitat Autònoma de Barcelona, Bellaterra, Catalunya, Spain

**Keywords:** repetitive DNA, transposable elements, satellite DNA, fiber-FISH, β-heterochromatin, polytene chromosomes

## Abstract

Transposable elements (TEs) and satellite DNAs (satDNAs) are abundant components of most eukaryotic genomes studied so far and their impact on evolution has been the focus of several studies. A number of studies linked TEs with satDNAs, but the nature of their evolutionary relationships remains unclear. During in silico analyses of the *Drosophila virilis* assembled genome, we found a novel DNA transposon we named *Tetris* based on its modular structure and diversity of rearranged forms. We aimed to characterize *Tetris* and investigate its role in generating satDNAs. Data mining and sequence analysis showed that *Tetris* is apparently nonautonomous, with a structure similar to foldback elements, and present in *D. virilis* and *D. americana*. Herein, we show that *Tetris* shares the final portions of its terminal inverted repeats (TIRs) with DAIBAM, a previously described miniature inverted transposable element implicated in the generation of chromosome inversions. Both elements are likely to be mobilized by the same autonomous TE. *Tetris* TIRs contain approximately 220-bp internal tandem repeats that we have named TIR-220. We also found TIR-220 repeats making up longer (kb-size) satDNA-like arrays. Using bioinformatic, phylogenetic and cytogenomic tools, we demonstrated that *Tetris* has contributed to shaping the genomes of *D. virilis* and *D. americana*, providing internal tandem repeats that served as building blocks for the amplification of satDNA arrays. The β-heterochromatic genomic environment seemed to have favored such amplification. Our results imply for the first time a role for foldback elements in generating satDNAs.

## Introduction

Repetitive DNAs are major components of most eukaryotic genomes studied so far, comprising more than 50% of the total DNA in many organisms, such as humans, maize, and the red flour beetle (Wang et al. 2008; Schnable et al. 2009; de Koning et al. 2011). They include many types of elements that can be classified based on their genomic organization as tandem or interspersed repeats.

Satellite DNAs (satDNAs) are the most abundant class of tandem repeats. They consist of repeat units typically organized as large arrays (up to several megabases), oriented in a head-to-tail fashion, and located in heterochromatic regions of chromosomes near centromeres and telomeres (Charlesworth et al*.* 1994; Plohl et al*.* 2008). Interspersed repeats are mainly represented by transposable elements (TEs), which consist of repeats that are able to proliferate and move throughout a genome (Kidwell 2002). TEs are classified in a hierarchical system of many levels. The first level is that of class and divides TEs into two groups based on the existence of an RNA intermediate step in transposition. Lower levels of classification include subclass, order, and superfamily (Wicker et al. 2007).

Several studies show that TEs have a more dispersed chromosome distribution compared with satDNAs, but both tend to accumulate preferentially in heterochromatic regions (Pimpinelli et al*.* 1995; Heslop-Harrison and Schwarzacher 2011). TEs and satDNAs are among the most rapidly evolving genomic elements and account for most of the variation seen in genome size and architecture among eukaryotes (Devos et al. 2002; Bosco et al. 2007).

The finding that some satDNAs share sequence similarity to TEs suggests an evolutionary link between these two types of repetitive DNAs. However, this issue was investigated only in a few studies. For example, data reviewed by Wong and Choo (2004) suggest that centromeric satDNAs may originate from whole or internal parts of TEs, probably by unequal crossing over (UCO). Most of such cases described so far involve retroelements from the gypsy superfamily in plants (Cheng and Murata 2003; Macas et al. 2009; Sharma et al. 2013). Studies of this phenomenon in animal species have evidenced no generalities. Examples include the *SGM* DNA transposons in *Drosophila guanche* (Miller et al. 2000), a Tc1-like element in the frog *Rana esculenta* (Pontecorvo et al. 2000), and the L1 retrotransposon in cetaceans (Kapitonov et al. 1998). Gaffney et al. (2003) also described a miniature inverted transposable element (MITE)-like interspersed repeat in *Crassostrea virginica* showing similarity to satDNA from several mollusk species. The identification of additional cases, specially using data from sequenced animal genomes, may contribute for a better understanding of the origin of satDNAs from TEs, the factors and mechanisms related to such phenomenon and its importance for genome evolution.

*Drosophila virilis* (*virilis* group; *Drosophila* subgenus) is one of the *Drosophila* species whose sequenced genome is publicly available (*Drosophila* 12 Genomes Consortium 2007). This species represents a particularly interesting model for the study of repetitive DNAs because it has one of the largest genomes (∼400 Mb) and one of the highest heterochromatic contents (∼50%) within the *Drosophila* genus (Mahan and Beck 1986; Bosco et al. 2007). As for most shotgun-sequenced eukaryote genomes, the *D. virilis* assembled genome mainly covers the euchromatic fraction. This hinders the characterization of repetitive DNA and requires the simultaneous employment of other methodologies.

More than 800 TE families have been identified in the sequenced genome of *D. virilis*, most of them long terminal repeat (LTR) or non-LTR retrotransposons (Feschotte et al. 2009). Together, they represent about 14% of the *D. virilis* genome (*Drosophila* 12 Genomes Consortium 2007). In spite of the large number of identified TEs, very little is known about their characteristics, such as detailed structure, copy number, genomic distribution, and association with other genetic elements. The recent availability of the sequence data for the genome of *D. americana* (Fonseca et al. 2013), another member of the *virilis* species subgroup (Morales-Hojas et al. 2011), further helps to study the repetitive DNA fraction in *D. virilis*, because it offers a phylogenetically close genome for comparisons.

In this study, we discovered a novel DNA transposon in *D. virilis* that we named *Tetris*. The name is a reference to the classic game TETRIS and alludes to the characteristics of the transposon, for example, its modular structure and diversity of rearranged forms. We used a combination of bioinformatic and experimental approaches in order to characterize *Tetris* and to discuss its role in generating a new satDNA in *D. virilis*. Our results imply for the first time a role for foldback DNA transposons in the generation of satDNAs.

## Materials and Methods

### Identification of *Tetris* in *D. virilis* and Sequence Analysis

We serendipitously found *Tetris* in the assembled genome of *D. virilis* while looking for tandemly repetitive DNA sequences using the Tandem Repeats Finder software (Benson 1999). The BLAST tool was used for searching sequences similar to *Tetris* in the 21 sequenced genomes of *Drosophila* available in FlyBase (http://flybase.org, last accessed May 31, 2014) and in the *D. americana* genome (available at http://cracs.fc.up.pt/∼nf/dame/, last accessed May 31, 2014). Hits with *e* values smaller than 10^−5^ and covering at least 60% of the query sequence were selected for further analysis. BLAST searches for annotated sequences were performed against the nucleotide collection (nr/nt) database in GenBank.

Sequence alignments were performed with Muscle (Edgar 2004) implemented in MEGA5 (Tamura et al. 2011) with default setup. MEGA5 was also used for the estimation of genetic distances (*p* distance), AT content, and repeat length analysis. The evolutionary relationships among sequences were inferred by neighbor-joining (NJ) trees using the implemented option in MEGA5 and the proportion of nucleotide differences (*p* distance).

### DNA Extraction, PCR Amplification, Cloning, and Sequencing

**Fig. 1.—:**
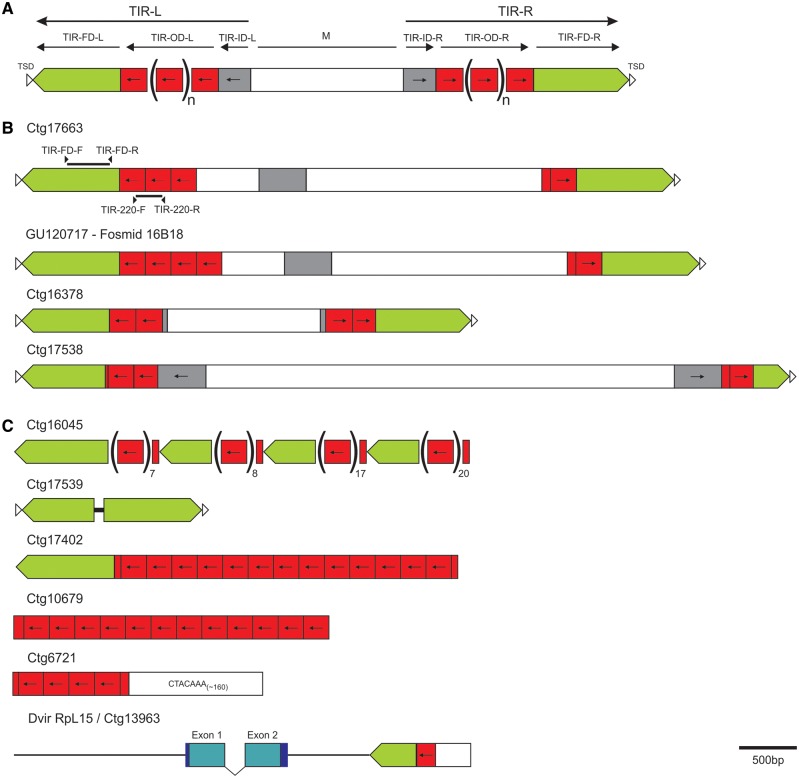
Schematic representation of the foldback structure and examples of the main types of variation found in the analyzed *Tetris* copies. (*A*) Schematic representation of foldback elements (adapted from Casals et al. 2005). M, middle domain. L and R refer to the left and right extremities of the transposon, respectively. (*B*) Schematic representation of the structurally complete copies of *Tetris* found in the *Drosophila virilis* assembled genome plus one hit from Fosmid 16B18 (accession: GU120717). Arrowheads in opposite orientation and solid black bars on top of the Ctg 17663 scheme represent the primers used to amplify the two fragments used as probes for the FISH experiments. (*C*) Main types of arrangements found between the *Tetris* and its TIR-220 repeats in the *D. virilis* genome, including one partial copy of *Tetris* next to the short heterochromatic gene RpL15. Green boxes represent *Tetris*’ TIR. Red boxes indicate the tandem repeats (TIR-220), and the grey boxes represent the *Tetris*’ TIR-ID.

Genomic DNA was extracted from a pool of individuals from the *D. virilis* strain 15010-1051.51 (Santiago, Chile). PCR amplification was carried out with two sets of primers: Primers terminal inverted repeat (TIR)-220-F (CACGATTTATCAATCATTTTGC) and TIR-220-R (CTCTATATGCACAACTACCGTGC) were designed to amplify the TIR-220 tandem repeats, whereas primers TIR-FD (flanking domain)-F (TCGAGCCCTTATTTCTTAGC) and TIR-FD-R (AACTGCGCTATTAAGTATGAC) were designed to amplify a part of the TIR-FD domain. The position of each primer is indicated in figure 1*B*. PCR products were purified and cloned using the pGEM-T-Easy cloning kit (Promega). Recombinant plasmids were sequenced on the ABI3130 platform (Life Technologies).

### Fluorescence In Situ Hybridization

Neuroblasts and salivary glands of third instar larvae from *D. virilis* (strain 15010-1051.51) and *D. americana* (strain W11) were used to obtain metaphase and polytene chromosome preparations, respectively (Baimai 1977; Ashburner 1989). Extended DNA fibers were obtained from adult flies and slides containing chromosomes or DNA fibers were prepared for fluorescence in situ hybridization (FISH) as described by Kuhn et al. (2008). The probes used for FISH were obtained from recombinant plasmids labeled with digoxigenin 11-dUTP or biotin 11-dUTP by nick translation with the DIG-nick and Biotin-nick translation mix (Roche Applied Science), respectively. Chromosomes were denatured by incubation in 0.07 M NaOH for 3 min and DNA fibers were denatured by incubation in 70% formamide/2× SSC at 80 °C for 3 min. The hybridization mix consisting of 100–200 ng of each probe in 50% formamide/2× SSC and water to a final volume of 40 µl per slide was denatured for 10 min at 80 °C and applied onto the slides. Hybridizations were performed in a moist chamber at 37 °C for 16–20 h. Posthybridization washes consisted of two baths in 2× SSC at 37 °C for 5 min. The probes were immunodetected with antidigoxigenin—FITC and neutravidin—Rhodamine (Roche Applied Science). Chromosomes and DNA fibers were counterstained with DAPI (4′,6-diamidino-2-phenylindole) in antifade reagent (SlowFade; Invitrogen) and analyzed under an Axio Imager A2 epifluorescence microscope equipped with the AxiocamMRm camera (Zeiss). Images were captured with Axiovision (Zeiss) and edited in Adobe Photoshop.

### Dot-Blot of TIR-FD and TIR-220 in *D. virilis* and *D. americana*

In order to detect and verify the abundance of sequences similar to TIR-220 and TIR-FD, genomic DNA of *D. virilis* and *D. americana* was dot-blotted onto a positively charged nylon membrane (Roche) and hybridized with cloned TIR-220 and TIR-FD probes separately. Control DNA DIG-labeled (Roche) and the probe plasmids were also dot-blotted as positive controls. DNA from *D. buzzatii* was used as a negative control. Positive signals were visualized using chemoluminiscent CDP-Star (Roche) and images acquired using the ChemiDoc XRS system and Quantity ONE 4.7 software (Bio-Rad) at the Laboratory of Analysis and Photodocumentation of the Universitat Autònoma de Barcelona.

## Results

### Characterization of *Tetris*

After genome searches for repetitive elements in the *D. virilis* assembled genome, we serendipitously found a TE with a structure very similar to that of foldback elements (see fig. 1*A*). Foldbacks are a group of DNA transposons first described in *D. melanogaster* (Potter et al. 1980) and later found in several other organisms, including rye, *Arabidopsis*, the sea urchin and *Chironomus thumi* (Lieberman et al. 1983; Hankeln and Schmidt 1990; Windsor and Waddell 2000; Alves et al. 2005). We named this element *Tetris*. Sequence analysis of *Tetris* revealed long TIRs (up to 1,735 bp) made up of three possible domains: A most external flanking domain (FD) (TIR-FD) with up to 866 bp, an intermediate outer domain (TIR-OD) with a variable number of approximately 220-bp tandem repeats (named TIR-220), and an inner domain (TIR-ID). The TIRs are separated by a middle domain (M) with variable composition and size and no apparent protein-coding capacity. We found only three “structurally complete” copies of *Tetris* (i.e., copies with conserved structure and identifiable target site duplications) in the *D. virilis* assembled genome (fig. 1*B*; supplementary table S3, Supplementary Material online). These three copies lack conserved open reading frames (ORFs) and showed 8–9 bp target site duplications (TSDs). Additional analysis of four rearranged copies of *Tetris* revealed TSDs between 9 and 10 bp (supplementary table S1, Supplementary Material online).

BLAST searches using the three complete sequences of *Tetris* as queries against the 21 *Drosophila* sequenced genomes available at FlyBase showed no significant hits, except for those observed in *D. virilis* in this study.

BLAST searches using the *Tetris* sequences against the nucleotide collection available in GenBank retrieved several hits from fosmids mapped on chromosome 6 (Müller element F, also known as the “dot” chromosome) of *D. virilis* (Leung et al. 2010). Most of these sequences consist of small fragments or rearranged copies of *Tetris*. One of them (from fosmid 16B18; accession: GU120717) showed an allelic copy of the *Tetris* element present in contig 17663 from the *D. virilis* assembled genome (fig. 1*B*). These two copies are nearly identical, except for the fact that the copy from the assembled genome has three TIR-220 repeats in the left TIR and the copy from the sequenced fosmid has four TIR-220 repeats. Sequence comparisons and phylogenetic analysis allowed us to infer that this array size variation was due to the duplication of the second TIR-220 repeat in the fosmid copy (supplementary figs. S1 and S2, Supplementary Material online).

**Fig. 2.—:**
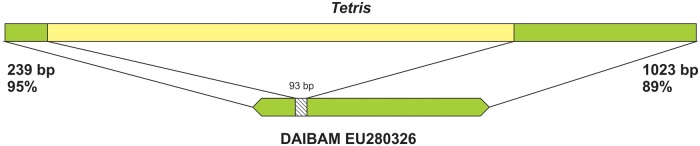
Schematic comparison between *Tetris* (Ctg 16378) and DAIBAM (EU280326). Regions with high sequence identity between the elements are shown in green. The DAIBAM region that could not be confidently aligned with *Tetris* is shown in the striped bar.

Besides the fosmids from *D. virilis*, BLAST searches in GenBank with *Tetris* as query retrieved significant hits in *D. americana*, another member of the *virilis* group. These hits correspond to an element that was found in the breakpoints of three chromosomal inversions and that was tentatively classified as a MITE and named DAIBAM (Evans et al. 2007; Fonseca et al. 2012). The similarity between *Tetris* and DAIBAM is high at both TIRs (left and right) of *Tetris* (95% and 89% identities) and actually extends over most of DAIBAM sequence (95%) (fig. 2). When self-aligned, DAIBAM copies show only short TIRs (up to ∼240 bp) and no further identifiable features. The extent of similarity between DAIBAM and *Tetris* indicates that DAIBAM probably originated from an element similar to *Tetris* by deletion (fig. 2).

### Characterization of TIR-220 Repeats

The TIRs from *Tetris* include an outer domain (OD) (TIR-OD) with a variable number of approximately 220-bp tandem repeats that we named TIR-220. BLAST searches using a TIR-220 consensus sequence against the *D. virilis* assembled genome retrieved 610 TIR-220 repeats distributed along 69 contigs. In 25 contigs, TIR-220 repeats are associated with adjacent TIR-FD segments of *Tetris* (*n* = 202), 39 contigs comprise exclusively TIR-220 repeats (*n* = 395), and five contigs encompass TIR-220 repeats neighboring other genomic elements (*n* = 13) but without the TIR-FD (supplementary table S2, Supplementary Material online).

A PCR experiment using primers with opposite orientations designed to amplify solely TIR-220 repeats resulted in a “ladder-like” pattern of amplicons representing multimers (up to seven) of approximately 220 bp, confirming the tandem organization of TIR-220 repeats seen in the assembled genome of *D. virilis* (supplementary fig. S3, Supplementary Material online).

Sequence analysis of 482 copies of TIR-220 repeats showed that they are AT-rich (∼70% on average) and highly homogeneous, displaying an overall similarity of approximately 94% (±0.4%) and an average repeat length of 218 bp. When analyzed separately, the TIR-220 repeats located inside structurally complete *Tetris* elements revealed a mean nucleotide similarity of 87% (±1.3%), whereas the rest of the TIR-220 repeats presented an average similarity of 94%. This result indicates that TIR-220 repeats are more homogeneous within long than within short arrays inside *Tetris*. Such pattern would be expected if a small number of copies within *Tetris* suffered expansion generating large TIR-220 arrays.

We found 27 complete out of 60 identified copies of TIR-220 in the *D. americana* assembled genome, but only as part of short arrays (up to three copies), typical for the TIRs of *Tetris*.

### Phylogeny of TIR-220 Repeats

**Fig. 3.—:**
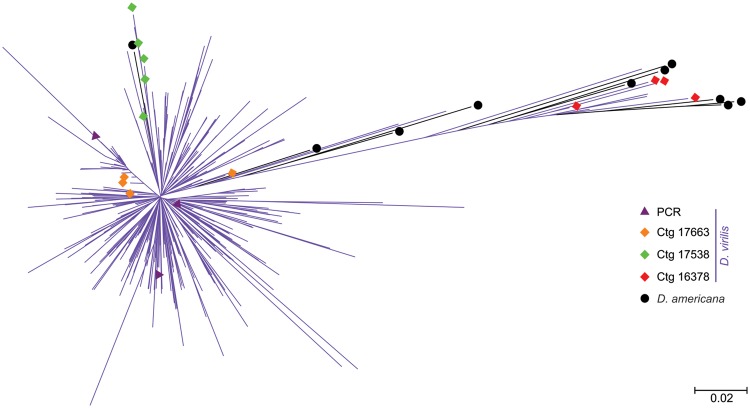
NJ tree containing TIR-220 repeats extracted from *Drosophila virilis* and *D. americana* assembled genomes. Purple branches with no symbols at the tips represent the bulk of *D. virilis* amplified copies. Repeats belonging to the three structurally complete copies of *Tetris* are shown in orange (Ctg 17663), red (Ctg 16378), and green (Ctg 17538) diamonds. Purple triangles represent the three *D. virilis* TIR-220 repeats obtained by PCR and sequenced in this work. *Drosophila americana* TIR-220 repeats are shown in black circles and branches. The tree was estimated using the NJ algorithm and the *p*-distance substitution method.

An NJ tree with all sampled TIR-220 repeats from *D. virilis* and *D. americana* revealed two main groups of sequences (fig. 3). The first one, on the left part of the tree, is mainly comprised short branches of TIR-220 repeats from *D. virilis* and a single TIR-220 copy from *D. americana*. The right side of the tree shows long branches leading to the repeats that are located inside one structurally complete copy of *Tetris* from *D. virilis* and to most repeats from *D. americana*.

The NJ tree showed no consistent clustering of TIR-220 repeats from single contigs, indicating that adjacent TIR-220 repeats are as similar to each other as they are to distant repeats present in the same or different arrays. However, in two of the three complete *Tetris* elements of *D. virilis* (Ctg 17538 and 16378) the TIR-220 repeats were grouped together, indicating the possible action of homogenization mechanisms (e.g., UCO and gene conversion). The TIR-220 sequences obtained by PCR are dispersed throughout the left side of the tree, indicating that they belonged to the larger and more abundant TIR-220 arrays (fig. 3).

### Chromosome Location of *Tetris* and TIR-220-bp Repeats

The karyotype of *D. virilis* consists of five pairs of large acrocentric chromosomes (Muller elements A–E) and a very small pair of microchromosomes (Muller element F; also known as dot chromosomes) and is thought to retain the ancestral condition inferred for the *Drosophila* genus (Clayton and Guest 1986). The karyotype of *D. americana* shows a centromeric fusion of chromosomes 2 and 3, and a polymorphic centromeric fusion of chromosomes X and 4 (Caletka and McAllister 2004).

In order to investigate the genomic location of both TIR-220 repeats within structurally complete copies of *Tetris* and expanded TIR-220 arrays, we performed double FISH experiments in metaphase and polytene chromosomes of *D. virilis* and metaphase chromosomes of *D. americana*. Probes for FISH were prepared from two regions of the *Tetris* element: One specific for the TIR-FD region and another specific for the TIR-220 repeats (fig. 1*B*).

**Fig. 4.—:**
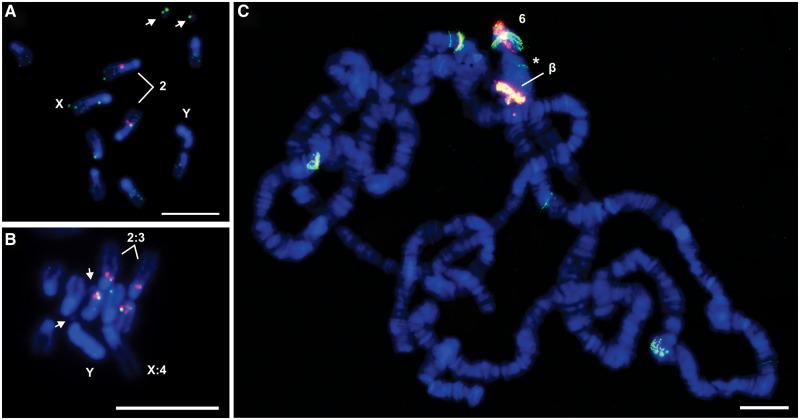
FISH of TIR-FD (green) and TIR-220 (red) probes hybridized on (*A*) *Drosophila virilis* metaphase, (*B*) *D. americana* metaphase, and (*C*) *D. virilis* polytene chromosomes. Arrows in (*A*) and (*B*) indicate the chromosome 6 (the dot chromosome). The chromocenter of the polytene chromosomes is indicated with an asterisk in (*C*). The “β” indicates the chromosome 2 β-heterochromatin and the number 6 is placed next to the polytene arm corresponding to the chromosome 6. Bars in (*A*) and (*B*) correspond to 10 µm, and in (*C*), to 5 µm.

FISH in the mitotic chromosome spreads of *D. virilis* produced colocalizing hybridization signals of both the probes in the distal extremity of the pericentromeric heterochromatin of chromosome 2 (Müller element E), identified as the biggest acrocentric autosome (fig. 4*A*). In this specific region, the signals corresponding to TIR-220 repeats were seemingly brighter than the signals produced by TIR-FD, suggesting overabundance of TIR-220 repeats. The two probes also colocalized in the small dot chromosomes, showing similar signal intensities. Small hybridization signals with the TIR-FD probe were distributed throughout all chromosomes, including the Y. The absence of such ubiquitous distribution of TIR-220 signals may be explained by the presence of *Tetris* elements containing partial or no TIR-220 repeats and therefore difficult to detect (see examples in fig. 1).

FISH in *D. americana* mitotic chromosomes with the same two probes produced colocalizing hybridization signals in the distal extremity of the pericentromeric heterochromatin of chromosomes 2 and 3 (Müller elements E and D respectively; fig. 4*B*). As observed for *D. virilis*, signals corresponding to TIR-220 repeats were seemingly brighter than the signals produced by TIR-FD. No hybridization signals were detected in the dot chromosomes of *D. americana*.

FISH experiments on polytene chromosomes of *D. virilis* using the same probes produced strong colocalizing hybridization signals with similar intensities in the proximal region of chromosome 2. This region consists of β-heterochromatin, an intermediate zone located between the heterochromatin and the euchromatin that, unlike α-heterochromatin, is replicated during polytenization. In the dot chromosome, strong colocalizing hybridization signals were observed in a region that extends from the end of the chromocenter to the most distal regions of the chromosome arm. A few euchromatic signals of TIR-FD with or without TIR-220 were also observed (fig. 4*C*).

The analysis of the assembled genome of *D. virilis* showed that some *Tetris* elements are present in contigs assigned to chromosomes 2, 3, and X. Interestingly, in two contigs (one of them is shown in fig. 1*C*) we found TIR-220 repeats ending abruptly with the Satellite I sequences of *D. virilis* (5′-ACAAACT-3′) (supplementary table S2, Supplementary Material online), known to comprise 20% of the genome and located in the tightly packed α-heterochromatin from all chromosomes (Gall and Atherton 1974) (fig. 1*C*). Such configuration further indicates that *Tetris* elements with expanded arrays accumulated close to the α-heterochromatin of chromosome 2.

### Long-Range Organization of *Tetris* and TIR-220 Repeats

**Fig. 5.—:**
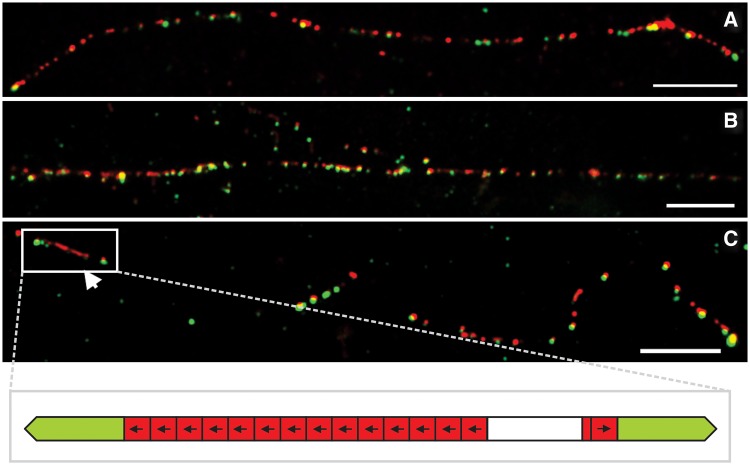
FISH of TIR-220 and TIR-FD probes onto extended DNA fibers of *Drosophila virilis*. Long DNA fibers with several *Tetris* hybridization signals including some expanded TIR-220 arrays are shown in (*A*) for *D. americana* and in (*B*) for *D. virilis*. Arrowhead in (*C*) indicates a long array of TIR-220 within *Tetris* from *D. virilis*. A representation of the hybridization signals highlighted in (*C*) is shown in the bottom row. The bars correspond to 10 kb (assuming 10 µm = 29 kb; Schwarzacher and Heslop-Harrison 2000).

High-resolution FISH on DNA fibers (fiber-FISH) with the two probes described above showed several cases where multiple copies of *Tetris* are located in a genomic region corresponding to less than 100 kb (fig. 5*A* and *B*). Such regions may correspond to those characterized by the strong hybridization signals observed in the metaphase and polytene chromosomes. The fiber-FISH results confirm the variety of rearrangements between *Tetris* and TIR-220 that we found in our in silico analyses of the *D. virilis* assembled genome (fig. 1*C*) and indicate a similar scenario for *D. americana*. In some fibers, it was possible to visualize TIR-FD hybridization signals associated with a variable number of TIR-220 repeats, including the presence of long arrays of TIR-220 repeats flanked by two TIR-FD (fig. 5*C*). No array composed solely by TIR-220 was detected by fiber-FISH. The presence of isolated signals generated only by the TIR-FD probe indicates the presence of *Tetris* elements containing none or partial TIR-220 repeats.

### Relative Abundances of TIR-FD and TIR-220 in *D. virilis* and *D. americana*

To gain further insight on *Tetris* abundance in *D. virilis* and *D. americana* genomes, we performed dot-blot hybridizations using the same TIR-FD and TIR-220 probes. For both species, the results indicated a marked overabundance of the TIR-220 sequences when compared with the TIR-FD probe, thus supporting the amplification of the former within *Tetris*. The abundance of TIR-220 in *D. americana* as evidenced by the dot-blot seems in contrast with the low copy number found in the assembled genome.

## Discussion

### *Tetris* Is a Foldback TE

In this work, we identified a TE in the assembled genome of *D. virilis* with structural similarities to foldback elements, which has been named *Tetris*. However, we found only three “structurally complete” copies of *Tetris*, that is, copies with conserved structure and identifiable target site duplications (fig. 1*B*), all of them lacking conserved ORFs and therefore defective or nonautonomous. Despite our efforts, autonomous copies encoding the transposase that mobilizes *Tetris* have not been found, perhaps because there are no complete copies in the sequenced *D. virilis* genome.

It has been suggested that the foldback structure may evolve independently from different lineages of TEs by the loss of the transposase gene and the elongation of TIRs (Marzo et al. 2008). However, most foldback elements seem to belong to the *Mutator* superfamily (Feschotte and Pritham 2007; Marquez and Pritham 2010; Gross and Williamson 2011), although there are exceptions (Marzo et al. 2008; Badal et al. 2013). The lack of a complete copy encoding the transposase prevents a definite classification of *Tetris* within the order of TIR transposons (Wicker et al. 2007). Nevertheless, the size of TSDs is related to the transposase that catalyzes the cut-and-paste reaction of TIR transposons and is an important clue that differentiates many of the TE superfamilies (Feschotte and Pritham 2007; Yuan and Wessler 2011). Based on the verified *Tetris* TSDs (8–10 bp) and from previous suggestions that foldback elements belong to the Mutator superfamily, we suggest that *Tetris* belongs to the Mutator superfamily.

### DAIBAM Is Closely Related to *Tetris*: Implications for Chromosome Inversions

It has been shown that *Drosophila* chromosome inversions may be generated by ectopic recombination between copies of the same transposon (Evans et al. 2007; Delprat et al. 2009; Fonseca et al. 2012; Rius et al. 2013). In *D. buzzatii*, for instance, three polymorphic inversions, *2j*, *2q*^7^ and *2z*^3^, were generated by the transposon *Galileo* that belongs to the P superfamily (Delprat et al. 2009). Likewise, *BuT5*, a MITE associated with the *P* element, generated two inversions fixed in the repleta group, *2s* and *2x*^3^ (Rius et al. 2013). Finally, a MITE named DAIBAM was involved in the origin of three chromosome inversions present in the *D. virilis* phylad, *Xa* fixed in *D. virilis*, and *4a* and *5a* polymorphic in *D. americana* (Evans et al. 2007; Fonseca et al. 2012). Herein, we show that the previously studied copies of DAIBAM are related to *Tetris* and present a high similarity at both ends of the element. Similarity between terminal sequences of a transposon and a MITE has been previously found and seemingly indicates that the autonomous element is responsible for the MITE origin and amplification because these sequences are known to be important for transposition (Rius et al. 2013). However, the available information on DAIBAM (Fonseca et al. 2012) is very limited and a more thorough characterization of its copies in the *D. americana* genome would be desirable. In addition, the complete element encoding the transposase that mobilizes *Tetris* and DAIBAM was not found. The characterization of such canonical copies would be also important for a definite classification of these elements.

### Origin of TIR-220 Repeats and First Stages of Copy Number Variation

**Fig. 6.—:**
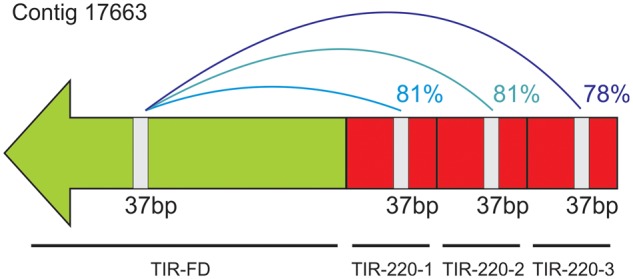
Sequence identity between a 37-bp stretch shared by the TIR-FD and TIR-220 domains of *Tetris*. The nucleotide identity values are given on the right side of the connection lines.

The TIR from *Tetris* is composed of a most external FD (TIR-FD), an intermediate outer domain with a variable number of tandem repeats (TIR-220), and an ID (TIR-ID). A careful comparison of the TIR-FD and TIR-220 domains revealed that they share a stretch of 37 bp with high similarity (∼80%) (fig. 6). This result suggests that the TIR-220 repeats may have originated as a duplication of a part of the original TIR-FD sequence. The foldback-like transposon *Galileo* of *D. buzzatti* also possesses internal tandem repeats within the TIRs that contain additional transposase binding sites (Marzo et al. 2013). These secondary binding sites may facilitate recognition by the THAP (Thanatos-associated protein) domain of the *Galileo* transposase. Increasing the number of tandem repeats containing the THAP recognition sequence may also lead to an increased transposition efficiency (Potter 1982; Cheng et al. 2000; Marzo et al. 2013). We suggest a similar scenario for the evolution of TIR-220 repeats from *D. virilis*. Initial stages of formation and expansion of the TIR-220 repeats may have increased the transposition efficiency of *Tetris* through the advantages explained above. However, larger arrays of TIR-220 may have disrupted the transposition of the *Tetris* element, leaving the door opened for the formation of satDNA arrays. Further studies will be needed to evaluate these suggestions.

UCO has long been considered as an important mechanism leading to the expansion or contraction of tandemly repeated DNAs, such as satDNAs (Smith 1976). In addition, rolling-circle replication of extrachromosomal repeats followed by reintegration in the genome may be another powerful mechanism for array expansion (Cohen and Segal 2009). Although there are several examples in the literature showing the participation of UCO in array size variation (Martin et al. 2003; Alkan et al. 2004), the participation of rolling-circle replication remains hypothetical. In this study, we found two allelic copies of *Tetris* that are nearly identical, except for the fact that one allele has three TIR-220 repeats and the other has four (fig. 1*B*; supplementary figs. S2 and S3, Supplementary Material online). We showed, by sequence comparisons and phylogenetic analyses, that this variation resulted from the duplication of the second TIR-220 copy present in one of the alleles. This duplication could be the outcome of an event of UCO between TIR-220 repeats. Nevertheless, other mechanisms may have also participated in the expansion of TIR-220 repeats inside *Tetris*.

### Large TIR-220 satDNA Arrays Originated Inside *Tetris*

During the analysis of the *D. virilis* assembled genome, we also found TIR-220 repeats as part of long and homogeneous satDNA arrays made of up to 66 copies (see supplementary table S2, Supplementary Material online). By a combination of sequence analysis and FISH on chromosomes and DNA fibers, we showed that TIR-220 repeats have undergone tandem amplification within *Tetris*. Such amplification may have resulted in the disruption of *Tetris* thus facilitating the expansion of large satDNA arrays.

Previous studies showed that TEs may provide different substrates for satDNA emergence. A few studies suggested that the first steps of satDNA formation could involve UCO promoted by ectopic recombination between TEs (Wong and Choo 2004). Consequently, some satDNAs share high degrees of sequence similarity to parts of the original TEs. Examples showing this phenomenon include the centromeric retroelements of maize (Sharma et al. 2013), the L1 retroelement of cetaceans (Kapitonov et al. 1998), the Ty3/*gypsy*-like retroelement from pea (Macas et al. 2009), and the *SGM* transposon in the *D. obscura* species group (Miller et al. 2000).

In this work, we show a different way by which TEs may provide templates for satDNA formation. In the case of the *Tetris* element of *D. virilis*, tandem repeat amplification involved repeats already present as part of the TE structure. It remains to be investigated if the peculiar repetitive structure of foldback-like elements makes them particularly prone to participate in the emergence of new satDNAs.

### *Tetris* Is Present in the *virilis* Subgroup

We found *Tetris* in two *Drosophila* species from the *virilis* group, that is, *D. virilis* and *D. americana,* suggesting that it was already present at least in the genome of the last common ancestor of the *virilis* subgroup*.* In the sequenced genome of *D. americana*, we found TIR-220 repeats organized only in short arrays made up of three repeats at most. Although this result suggested that no expansion of TIR-220 repeats took place in the *D. americana* genome, fiber-FISH and dot-blot analysis indicated otherwise.

Fiber-FISH results for *D. americana* were much alike those obtained for *D. virilis*, showing clusters of *Tetris* insertions with expanded internal TIR-220 arrays. Moreover, the dot-blot analysis revealed a similar abundance of TIR-220 in *D. americana* and in *D. virilis* (supplementary fig. S4, Supplementary Material online), suggesting that amplification of TIR-220 repeats also occurred in the first species. The much lower TIR-220 copy number in the *D. americana* assembled genome is probably a consequence of the shorter read lengths produced by next generation sequencers, what poses even bigger challenges to the assemblage of repetitive regions.

Based on our data, TIR-FD and TIR-220 repeats account for 0.08% of the assembled genome of *D. virilis*, but only half of its estimated genome size has been assembled so far. Additionally, repetitive DNAs represent a challenge for the assembly of genomes sequenced by the whole-genome shotgun methodology, such as that of *D. virilis* (*Drosophila* 12 Genomes Consortium 2007). In fact, most of the heterochromatic sequences from the *Drosophila* sequenced genomes remain unassembled and therefore the actual contribution of *Tetris* for the genome size of *D. virilis* may be higher.

### The Amplification of TIR-220 Occurred in the β-Heterochromatin

Multiple copies of *Tetris* were found scattered along all chromosomes of *D. virilis* and *D. americana*, but with a much higher density in some sites such as in the dot chromosome and β-heterochromatin of chromosome 2 of *D. virilis* and in the β-heterochromatin chromosomes 2 and 3 of *D. americana*. Such a distribution may indicate that part of the euchromatin of the dot chromosome shares common sequences with the β-heterochromatin of chromosome 2. This situation is similar to that found on *D. melanogaster*, in which the β-heterochromatin of the X chromosome and part of the euchromatin of the dot chromosome are composed of a shared mosaic of middle repetitive elements (Miklos et al. 1988). Interestingly, Slawson et al. (2006) also found an enrichment of repetitive elements in the dot chromosomes of *D. virilis* compared with the euchromatic parts of the remaining chromosomes. The heterochromatic accumulation of TEs is probably a result of the lower levels of recombination in this region. Once inserted in the heterochromatin, TEs are less prone to generate unviable rearrangements through ectopic recombination, what ultimately reduces the effectiveness of purifying selection upon these sequences (Petrov et al. 2011). Nevertheless, the mainly euchromatic *D. virilis* dot chromosome was shown to recombine at meiosis, although at a lower level. Some authors suggested that there might be an adaptive advantage in maintaining a high density of repetitive elements in the dot chromosome (Slawson et al. 2006). The dot chromosomes of *D. americana* seem to be devoid of *Tetris*.

The β-heterochromatin of chromosome 2 of *D. virilis* and chromosomes 2 and 3 of *D. americana* were the sites where major events of amplification of TIR-220 repeats took place. A more detailed study of the β-heterochromatin of *D. melanogaster* chromosomes revealed several interesting features including a high density of TEs and a high proportion of rearranged and mostly defective TEs (Vaury et al. 1989). In particular, sources of TE rearrangements in the β-heterochromatin include tandem amplifications and nested insertions (Vaury et al. 1989; Kuhn and Heslop-Harrison 2011). Such properties of β-heterochromatin add valuable and testable information related to the potential origin of satDNAs in this region.

## Conclusions

In this study, we showed by a combination of bioinformatic and experimental data that a foldback element, named here as *Tetris*, has contributed to shaping the genomes of *D. virilis* and *D. americana* by providing internal tandem repeats that acted as “seeds” for the amplification of satDNA arrays. The β-heterochromatin genomic environment associated with the high density of *Tetris* copies seemed to have favored such amplification. There is increasing evidence for the participation of different families of TEs in the origin of other types of repetitive DNAs, such as microsatellites (e.g., Wilder and Hollocher 2001; Smýkal et al. 2009), minisatellites (e.g., Inukai 2004), and satDNAs (e.g., Macas et al. 2009). Our results imply for the first time a role for foldback elements in the generation of satDNAs.

In *Drosophila*, transposons have been shown to generate chromosome inversions by ectopic recombination between copies of the same family, as is the case of the transposon *Galileo* in *D. buzzatti* (Delprat et al. 2009). In *D. americana*, a TE classified as MITE and named DAIBAM was found to be implicated in the generation of three chromosome inversions of the virilis phylad (Fonseca et al. 2012). Interestingly, we showed in this work that DAIBAM is related to *Tetris* by the sequence similarity present between their ends, a region known to be important for transposition. DAIBAM and *Tetris* are likely to be mobilized by elements of the same family and DAIBAM could have been generated from an element similar to *Tetris* by deletion of internal sequences.

## Supplementary Material

Supplementary tables S1–S3 and figures S1–S4 are available at *Genome Biology and Evolution* online (http://www.gbe.oxfordjournals.org/).
